# Hospital and Institutional News

**Published:** 1911-12-16

**Authors:** 


### HOSPITAL AND INSTITUTIONAL NEWS.

### LATEST AWARDS OF THE KING'S FUND.

From the Emperor's Camp, India, dated December 9, have arrived the congratulations of King George upon the distribution of awards by King Edward's Hospital Fund, of which he is patron. The full list appears on another page of this issue, and, at a time when the existence of the voluntary hospitals is endangered by rash legislation, peculiar significance attaches to his Majesty's congratulations on the increase of £2,500 in the annual amount at the disposal of the Fund for distribution. Inevitably the awards themselves excite mixed feelings among those institutions allotted, or not allotted, a place in the long list. But any heartburning would be cured at once if the penalty for grumbling were to place on the institution the task of distribution. Which of us would care to have the responsibility of the distribution committee; which would have the temerity to claim greater care and impartiality than are shown in its awards? Sir William Church as chairman, deserves the most sincere congratulations on his committee's achievement. The chief facts it has to record in its report are the disappearance of St. George's Hospital from the list of awards, owing to the conditions attached to all similar grants by the council of the Fund not having been fulfilled, and the delayed inception of an amalgamation scheme, which the Fund would desire to see completed, among the five London hospitals for the diseases of the throat, nose, and ear.

### HOW THE HOSPITALS ARE CRGANISING.

The policy of organisation recommended in The Hospital has been promptly taken up, and the voluntary hospitals are bestirring themselves to command the attention of their local members of Parliament, and to explain to their working-class supporters the "grievous injury" which the insurance scheme, unamended, will inflict. From York, from Lincoln, and from Liverpool, to take the first three letters from the batch we have received, news comes of hospital activity. In Lincoln, Mr. Arthur Moore, the secretary-superintendent of the County Hospital, has "acted on the instructions of his weekly board" and tackled the local member. In York, Mr. Fred Neden, the secretary and manager of the York County Hospital, has stirred the local member and the local Press as well. In Liverpool, the superintendent of the Royal Infirmary, Mr. F. H. Moore, has been directed to press the resolutions of the British Hospitals Association on "all the members of Parliament for this district." These three centres typify the activity of the hospitals at thirty others, and few hospitals, we believe, are left to enjoy the unenviable distinction of doing nothing. Much more has yet to be done. The working-men in every district must have the probable effects of the Bill, and its absence of provision for hospital treatment explained to them by the hospital secretaries and committee-men. A record of those hospitals which are first in the field in this awakening of the working-classes is essential, and we ask every hospital secretary to write to us as soon as he and his board have struck the first blow, and addressed the first meeting.

### SPECIAL INTERVIEW WITH DR. COLLINGRIDGE.

We publish this week the first instalment of an interesting interview with Dr. Collingridge, the retiring medical officer to the City. With great courtesy and in admirable fulness, Dr. Collingridge has summarised the progress of public health work at the Port of London as he has seen it develop, and indeed been personally responsible for it, during some part of his thirty-two years of public work. The Port of London is, comparatively speaking, a new institution, the importance of which is vaguely apprehended, but the pioneer work of which has been generally misunderstood. Dr. Collingridge lifts the curtain this week and reveals how the public health work of the Port has been built up. Next week we shall publish his striking résumé of the ground covered by the Public Health Department of the City Corporation, the multitudinous activities of which are symbolised every Lord Mayor's Day in a pageant as varied as themselves.

### OUT-PATIENT DEPARTMENT REORGANISED AT LEEDS.

As The Hospital has led its readers to expect, the ophthalmic and aural departments at the Leeds Infirmary are to be split into two, each with two honorary surgeons, while the work of the present throat department will be transferred to a new creation, an amalgamated ear, throat, and nose department with its own surgical staff. Thus there will be in future the three departments: the eye; the ear, throat, and nose; and the gynæcological. Both in and out patients will be attended in these special departments, but no assistant honorary officers are to hold appointments in them, as the present rules permit not more than twenty years' service for all full surgeons on the infirmary staff. In future the honorary officers will be elected for five years only, if re-elected at the end of that time for twenty years, or until the age of sixty. It is understood that Mr. H. Secker Walker and Mr. A. L. Whitehead shall be the first surgeons to the eye department without election, and that Mr. Constable Hayes shall be the first surgeon to the ear, throat, and nose department. The effect of these changes is that the ophthalmic and aural departments in future will be staffed by specialists.

### RAGGING AN ANATOMICAL LECTURER.

Professor Nicholas, Lecturer on Anatomy in the Medical School of the University of Paris, has had to interrupt his course, says the *Times* correspondent, on account of the ragging to which he has been subjected by the students at the Sorbonne. The facts that certain new examination regulations are unpopular and that Professor

Nicholas is "a member of the rival University of Nancy" are supposed to account for the disturbances.

### THE BOSTON HOSPITAL PROMISES REFORM.

The first meeting of the General Committee of the Boston Hospital since its annual meeting of subscribers was principally concerned with The Hospital's Report (November 4, page 131) on the condition of the institution. Mr. W. Porter, having been re-elected the Chairman, referred to the "piecemeal" manner in which the hospital had been erected, to the difficulties that resulted from this, and to the need for increased support, whereupon the Rev. Canon Heygate jumped to his feet and exclaimed "Whether our financial position will allow us to do anything or not, a report of this sort comes with authority and we ought not to pass it by." Canon Heygate dilating on our strictures concerning the accommodation for the staff, and said the matron had admitted to him that on Monday morning the nurses who were not on duty had to sit in the kitchen while their room was occupied by the House Committee. "Surely," asked the Canon, "it is unsatisfactory that the nurses should have beds in which practically they cannot sleep? Lastly," the speaker continued with emphasis, "those of the Committee who have been in the mortuary cannot help feeling that the criticism of it is very just. I propose that a sub-committee be formed to bring the matter before another meeting of the General Committee." The Rev. F. J. Brown seconded. Dr. R. Tuxford spoke to the same effect. Finally, Mr. W. Porter, Canon Heygate, the Rev. F. J. Brown, Dr. South, Mr. J. M. Simpson, and Mr. A. Cooke-Yarborough, secretary to the hospital, were appointed members of a sub-committee to consider the matter.

### HOW THE CLERGY OVERRODE OPPOSITION.

Dr. Tuxford was perfectly right in showing up the shallowness of alleging that all the faults of the institution were due to want of money. Such an excuse, as Dr. Tuxford evidently knew from experience, can be offered to justify any infamy. Indeed, it is only infamies which are bolstered upon it. The appointment of a sub-committee, with the vague object of considering a matter which is already understood as indefensible, is not very much, but even this would not have been gained had not the local clergy and one member of the medical staff insisted upon it. To Canon Heygate and Mr. F. J. Brown must redound the credit of courageously admitting the facts and insisting on their recognition. Such action will do more for their own reputation as public-spirited men, and more for their profession, than any hospital chaplain has done of recent years.

### SIR GEORGE LEWIS AS HOSPITAL OFFICER.

It may not be generally known that the late Sir George Lewis, whose famous reputation as a solicitor and confidential adviser to innumerable well-known people, whom indiscretion, bad luck, or the blackmailer had got into awkward positions, was a hospital officer in the sense that he had been appointed honorary solicitor to the King's Sanatorium. When a man has become identified so closely in the public mind with one particular form of activity, it is interesting to recall any fact about him which touches upon a widely different branch of work. Fortunately for the institution with which he was connected, as Sir George Lewis himself would have been the first to grant, his services remained honorary in the best sense, that the sanatorium never has been troubled seriously with litigation.

### A MODERN HOSPITAL COMMITTEEMAN'S WORK.

The public is so little aware and sometimes so sceptical of the practical work demanded of a hospital committeeman that we take the just made resignation of Mr. G. K. Morice from the Committee of the London Hospital as a practical example of up-to-date committee work, which also happens to throw a historical sidelight on the relations between the London Hospital and the Grocers' Company. By an agreement dated June 4, 1873, this company has the right to nominate two members of its Court to serve on the London Hospital's Committee, in consideration of its help in building what is still called the Grocers' Company's wing. Mr. G. K. Morice, who has passed the chair of the Grocers' Company, has been one of the company's two representatives on the Hospital's Committee since 1891. We have means of knowing that he was one of its most regular and energetic members. Thus for a long time he was Chairman of the Stewards' Committee, in virtue of which he had to take charge of the meat, milk, bread, and grocery contracts. His practical experience of this expert work makes his resignation a genuine loss to the London Hospital, and should serve to show how active a standard this hospital demands of its committeemen. Fortunately, such a standard is pretty general nowadays.

### THE SIN OF OMISSION IN MISSIONARY WORK.

The necessity for the elementary medical training of missionaries is alluded to, if still somewhat perfunctorily, in the latest report of Livingstone College. The report admits that elementary medical training hitherto has been regarded as the supererogatory "finish to a missionary education" rather than as the paramount necessity which it is. This is a fallacy which does not die, and which, the report naïvely goes on to remark, "has cost much to missionary societies in men and means." We are glad of this, even though it sounds hard to say so; partly because no mention is made of the loss which ignorance of medicine has caused to the people to whom the missionaries have been sent, but chiefly because missionaries should have recognised the duty they owed to science and research in their expeditionary activities, and also because the worst blunders with which this class of explorer can be credited—blunders which no missionary could wish to see repeated—might have been mitigated had even the slenderest acquaintance with medicine and anthropology been shown by the latest apostles of the Church.

### WHERE MISSIONARIES CAN BE TRAINED IN MEDICINE.

We are glad therefore to note the recent appointment of Dr. C. F. Harford, M.D., Principal and Secretary of Livingstone College, to the secretaryship of the Medical Missions of the Church Missionary Society, to which he was formerly physician; and to observe the great help which the Seamen's Hospital, Greenwich, the Walthamstow Hospital, through Dr. Frank Collins, Dr. Wise, and Dr. A. H. Bars, and the Poplar Hospital have given in allowing the Livingstone College students to see, at least, the elementary practice of medicine and surgery. The worst sin of missionary enterprise—the sin of omitting all study of medicine—will, perhaps, soon become a sin of the past.

### WOMEN DOCTORS IN INDIA.

Dr. Emma Slater, L.R.C.P.Ed., hon. secretary of the United Kingdom Branch Association of Medical Women in India, is agitating for a properly officered body of women doctors under State supervision, for, in fact, a Women's Indian Medical Service. She says, "The Government of Bombay alone saw the necessity of placing women doctors upon a proper status, and through the wisdom of the then Governor, Lord Reay, and the devoted labours of Dr. Edith Pechey, the Cama Hospital was inaugurated twenty-five years ago, the only Government hospital in India officered entirely by women, and it still remains a model of what a women's hospital should be."

### MEDICINE'S DEBT TO SIR JOSEPH HOOKER.

Of that group of distinguished and veteran medical men which includes Lord Lister, Sir John Kirk (of Zanzibar), Sir George Bindwood, the late Sir Joseph Dalton Hooker, who died on December 10, was the doyen. When it is remembered that the deceased scientist was surgeon on the *Erebus* to Sim James Ross's Antarctic Expedition in 1839, his great age of ninety-four is of special interest, for that expedition to the regions since again made famous by Shackleton and Scott set out more than seventy-two years ago. Then, again, it is to be recalled that when Darwin first conceived his ideas of the origin of species and the theory of evolution, it was to Hooker that he unburdened himself, and at Hooker's advice and instigation that he devoted himself to working out his discoveries. Besides achievements such as these, his twenty years' directorate of Kew Gardens is of little interest, though the fact that he succeeded his father in that post and was succeeded by his son-in-law may be of moment to students of heredity. The mere recital of his orders and distinctions shows in what esteem his scientific accomplishments were held: O.M., G.C.S.I., M.D., LL.D., F.R.S., and President of the Royal Society for five years are perhaps the chief. As an explorer in the Himalayas, Syria, Morocco, California, the Rocky Mountains, at dates when travel really was an arduous undertaking, Sir J. D. Hooker's exploits would have entitled him to fame. With him the medical profession loses one who has brought it nothing but honour.

### SIR EDGAR SPEYER ON HOSPITAL BENEFIT.

Not only was the record amount of some £3,000 subscribed at the annual festival dinner of the West Ham Hospital on Friday last week, but Sir Edgar Speyer, the Chairman, took most admirably the chance afforded to him by emphasising the disaster which the present unamended Insurance Bill threatens to inflict on the hospitals, including the one which serves West Ham. Sir Edgar Speyer urged the necessity of providing hospital treatment on an adequate scale for insured patients, and pointed out that those who understand the true value of the voluntary system must make the most devoted efforts to maintain it, or an indifferent public and a meddlesome Government will attempt to replace it with State administered hospitals. "The voluntary system," said Sir Edgar, "is an ideal one," and in its name West Ham Hospital secured a record collection. The hearts of Mr. T. A. Cook, the active Chairman, and of Mr. Scrivener, the secretary, must be temporarily relieved and both these gentlemen are to be much congratulated upon this result.

### ROYAL BUCKS HOSPITAL, AYLESBURY.

This hospital was inspected by H.H. Princess Victoria of Schleswig-Holstein on the 12th instant. Her Highness was received by the President, Mr. Leopold de Rothschild, C.V.O., and made a thorough inspection of the buildings, which have been modernised and brought up to date at a cost of nearly £12,000. The reconstruction is a great improvement in every way, and has made this hospital well up-to-date in several respects. The new buildings contain accommodation for a better class of patients who can afford to pay moderately for their accommodation and treatment. These additions have proved most popular and useful, and promise to be of the greatest comfort and help to members of the middle classes, who have heretofore been unable to secure such benefits. The institution still needs a Nurses' Home, and until this is provided some of the special accommodation just referred to is not available, owing to the necessity of providing members of the nursing staff with sleeping accommodation in the pay wards. A few years ago Mr. James Berry, F.R.C.S., was appointed consulting surgeon to this hospital, and since then he has rendered most material help and assistance to the medical staff. By acting in cooperation with them the work of the hospital has progressed materially in every way. There is now no excuse for residents in the county of Bucks to send patients to the London hospitals, for the Royal Bucks Hospital affords them every facility for excellent treatment with adequate guarantees of the best possible results. This hospital owes its present admirable equipment and efficiency in no small degree to members of the Rothschild family, who have rendered and are still rendering the most generous financial and personal help to this hospital.

### H.H. PRINCESS VICTORIA OF SCHLESWIG-HOLSTEIN.

H.H. Princess Victoria possesses the Royal gift in high degree of making herself most agreeable, whilst she takes the deepest interest in everything which tends to promote the comfort and recovery of the sick and injured. No time seems too great for the Princess to give to this beneficent work. Her inspection of the Royal Bucks Hospital proved once more how impressed Her Highness is with the privilege of personal service in the days of health. Not only did she inspect the wards and various departments of the hospital, but she was careful also to visit the quarters of the nurses and the general staff. Mr. Leopold de Rothschild paid eloquent testimony to the value of the princess's work, the gracious way in which it was invariably undertaken, and the successful results which had attended Princess Victoria's efforts on behalf of the hospitals through the League of Mercy. The four counties of which she is lady President, Bucks, Berks, Hants, and Oxon, have already raised £11,926, resulting in grants to hospitals in the four counties of £4,565. In six years Bucks has contributed to the League of Mercy £1,764, while grants have been made to the county and other hospitals by the League which collectively amount to £405.

### A SUCCESSFUL COUNTY CHAIRMAN.

The Royal County Hospital is situated in the district of Mid-Bucks, of which Mrs. Lee, of Hartwell, is the devoted lady Vice-President. The total sum raised by this branch is £188, but the Royal Bucks Hospital has received £260 in grants. The grants made in 1911 were £110, the largest sum yet given to the hospitals of Bucks. There was therefore abundant justification for the high testimony borne by Mr. Leopold de Rothschild, who is to be congratulated on the modernising of the Royal Bucks Hospital, to which his family and himself have contributed so materially by personal service and otherwise. The inhabitants of Bucks owe special thanks to Mr. George Carrington, the Chairman of this hospital, who has been as untiring as he has proved successful in the efforts he has made to bring his institution successful through this crisis, and to make it worthy of the best traditions of an English voluntary hospital.

### THE MANAGEMENT OF ST. KATHARINE'S HOSPITAL.

It is generally stated that the administration of St. Katharine's Hospital, Regent's Park, on the allegations against which we commented a few weeks ago, is now being carefully considered by Queen Alexandra, who is, of course, vested with the patronage of the institution. The mastership, now vacant, is therefore not likely to be filled until the whole question of the management of the funds has been considered.

### RECURRENT INSANITY IN MENTAL HOSPITALS.

The London County Council has rarely been heard to better advantage than at its meeting on December 5, when a really remarkable debate took place on the prevention of the increase of the feeble-minded. The Asylums Committee reported that a female patient who was admitted to Colney Hatch Mental Hospital in September, aged sixty-seven years, had been a patient at the same hospital on sixteen previous occasions. She has also been in other mental hospitals on eleven occasions. She was, moreover, married at eighteen years of age, and has had thirteen children, five of whom are dead. Her first attack of insanity was at the age of twenty-one, when her first child was born. The patient's children were said to be all healthy, with two exceptions. She had two aunts in mental hospitals and two of her sisters had suffered from insanity, one having died in Colney Hatch Mental Hospital some years ago and the other having committed suicide by hanging. The case seemed to the Committee a striking illustration of the need for legislation to prevent the multiplication of the unfit. Mr. A. O. Goodrich, chairman of the Asylums Committee, said that this was no isolated illustration of recurrent insanity, which the Asylums Committee would like to do something practicable to put a stop to.

### A HOT DEBATE ON DRASTIC MEASURES.

The problem of constantly readjusting these patients is causing the Committee a great deal of anxiety. At present it has no power to do anything, although the Committee knows very well indeed that when it discharges a patient there is every probability of his return. Mental hospital committees have no power whatever to detain a patient when once he is apparently recovered. Members of the Council, said Mr. Goodrich, who were not members of the Asylums Committee, would be rather astonished to know that during the years 1895 to 1910 no less than 29 per cent. of the patients who were discharged were subsequently re-admitted to the mental hospitals. It was very difficult to make practical suggestions in the matter; and it was for that reason that no recommendation had been brought forward. One suggestion which had been made to prevent recurrent insanity was segregation, and in this matter he commended the work done by the Eugenics Society, which was promoting a Bill in Parliament. Another suggestion was the colonisation, at such a spot as Lundy Island; another for a surgical operation, which would have to be under proper safeguards. The hereditary character of the disease had been proved beyond doubt. If the After-Care Association for poor persons discharged recovered from mental hospitals supports these views it is worth helping. How many, it is pertinent to ask Mr. H. Thornhill Roxby, of those he assists are really recovered and not recurrent cases?

### LEGAL STERILISATION OF THE INSANE.

In the discussion on sterilisation which followed Mr. Edward Smith and Sir John MacDougall were succeeded by Mr. H. H. Gordon, who said that if the religious "conscience" of the whole community is against sterilisation as the only and effective preventive method, the religious community itself should be made responsible for dealing with the problem. Mr. E. G. Easton said that, if sterilisation was legalised, at least 10 per cent. of the existing inmates of their mental hospitals would be perfectly free to be allowed out. Mr. A. A. Thomas, who quoted statistics from a report of the Asylums Committee, said that if the Council did its duty, in the next few years they would see a great drop in the number of inmates in the mental hospitals. Then Mr. H. V. Rowe said that it would be a very difficult matter to educate the public up to sterilisation, whereupon Mr. J. W. Gilbert said that if any attempt was ever made on the part of the Council to adopt some of the measures just advocated it would receive strong opposition from him at least. We have quoted this discussion at some length, not only because of its interest to mental hospital workers, but also as showing a startling unanimity and frank determination to grapple with the problem of recurrent insanity by direct interference at its root.

### THE INSECURITY OF MEDICAL OFFICERS OF HEALTH.

A serious grievance suffered by Medical Officers of Health was alleged by a speaker at the Mansion House Council on Health and Housing last week. Insecurity of tenure, it was stated, hampers the progress of medical officers' work at many points. The builders on the local councils, said the speaker, are so influential that the medical officers are often deterred from making adverse reports on property for fear of offending this powerful interest. Not dissimilar complaints have often reached us, and Medical Officers of Health all over the country would be well advised to send us their views on this matter. In this way a channel would be provided for the expression of united professional opinion, which is the first step towards inquiry and a practical scheme of reform.

### A LONDON FIRM'S GIFT TO HOSPITALS.

The opening of Whiteley's new buildings in Queen's Road last week was interesting to the institutional world from the fact that £500 was presented by the firm to Sir T. Crosby, the Lord Mayor, for distribution to London charities. The names of those selected have now been published, and show that St. Bartholomew's Hospital receives £200, the City of London Hospital for Diseases of the Chest, Victoria Park, £100, together with £100 each to the Royal London Ophthalmic Hospital in City Road and to Treloar's Home for Cripples. The great firms and their employees have always taken such a large share in supporting the voluntary hospitals that it is a very graceful act to memorise their own growth and extension by a special gift to voluntary institutions.

### PERSONAL SERVICE FROM FORMER PATIENTS.

The North Ormesby Hospital, Middlesbrough, which has a debt of £5,000, has received an offer of the added sum of 10 per cent. on all donations received before the 31st of the present month, and urges that the house to house collection be undertaken by many previous patients, so that the adequate number of collectors necessary be recruited and further reinforced by those who know better than anyone else the value of the institution to the sick. We hope that Mr. E. C. Smith, the secretary of the hospital, will find a large response, for there is little doubt that the most enthusiastic collectors will be those who have experienced the advantages of the institution for which they are asking support. The duty of ex-patients does not end with the subscription many of them give on leaving, and the appeal for their personal service at this juncture should attract the support of all who have in them a spark of gratitude for their recovery.

### INSTITUTIONAL TREATMENT IN THE ARMY.

The annual report on the health of the Army, in a paragraph dealing with institutional treatment, shows that 20.4 beds per thousand men have been constantly occupied during the past year. This is a number absolutely out of proportion to the number of beds available for those who will come under the Insurance Act, even if all hospital and infirmary beds were included. No doubt in the Army many conditions are treated in hospital which would never be "hospitalised" in civil life. It is more economical for the authorities to put the men in hospital and get them well at once, than to have sick men in barracks. But even allowing for this, the figures are very striking. Another point that these figures bring out is the great variability in the sickness ratio among different units. The most striking variations are seen among the men employed at the various regimental depôts, who may presumably be considered to be doing similar duties. The ratio per thousand varies among these men from 75 to 445, and these variations cannot be explained upon the difference between city and country town life, for some of the biggest numbers come under the latter. There must be some personal factor, or habit to help explain these figures. Indeed this is further seen from the figures of the Foot Guards, who have the greatest sickness ratio in the service with 34 men per thousand constantly in hospital, and at their depôt at Caterham 611 men were in hospital during the year out of 714, or a ratio per thousand of 855. That locality does, however, have some effect, is seen from the figures of the different commands. The London district has the highest ratio of 604, and the Western command the lowest with 245. All these and many other points are raised by the figures shown in this report, and must be applied to civil life in considering any scheme which professes to benefit the health of the masses.

### A STANDARD SYSTEM OF PRESCRIBING.

It is stated in practically all text-books on "dispensing" that quick dispensing is good dispensing, but one wonders if these experts meant this expression of opinion to be an encouragement to hospital dispensers. By this one does not mean to imply that good dispensing is absent in these departments; on the contrary the dispensing in large dispensaries is generally of a very high character, and most pharmacists are proud of their work. It will be readily acknowledged that in an out-patient department it is necessary that the work should be performed as quickly as is possible for good work to be, and when a man has dispensed for some considerable time he becomes tired and it is then he may overlook some slight differences in the prescriptions, but it abounds to the credit of pharmacy that it is very unusual for a mistake to be made. There are times when a prescriber adopts two different methods of writing his scripts. It would be better for both medicine and pharmacy if a standard system could be adopted.

### THIS WEEK'S DRUG MARKET.

Business in the drug market continues fairly brisk considering the near approach of the stock-taking period, and a number of price changes have taken place. A considerable advance in the price of opium is announced from Smyrna, and the extreme price quoted is likely to have some effect on the demand; there seems little probability of any decline in value for some months to come, a further advance, moreover, being quite probable. Morphine is also dearer, and a further rise in makers' prices of codeine is not unexpected. The extreme prices quoted for opium and its alkaloids naturally render caution desirable, but, in view of the scarcity of the raw drug, it might not be altogether unwise to procure a supply of these three articles sufficient to last for two months or so. Quicksilver has been reduced in price, and as a consequence makers of calomel, corrosive sublimate, and other salts of mercury have announced a reduction in their quotations of three-halfpence per lb. Apiol is considerably dearer, owing to scarcity of the raw material caused by the drought which prevailed in the summer. Japanese refined camphor tends higher in price, and an advance in the value of English refined would not come as a surprise. Lactic acid is dearer. Castor oil tends slightly lower in value. Balsam copaiba is still inclined towards dearer rates. The lower price at which cloves are selling has resulted in a reduction in quotations for clove oil. Sandalwood oil is dearer. Cod-liver oil is again rather lower in price. Best Jamaica sarsaparilla is wanted, and prices are likely to be higher. The decline in the value of sugar of milk has been arrested, and it is not anticipated that any notable reduction in price will take place at present. The quinine market is quiet again, and the price is unchanged.

